# COPD phenotype description using principal components analysis

**DOI:** 10.1186/1465-9921-10-41

**Published:** 2009-05-29

**Authors:** Kay Roy, Jacky Smith, Umme Kolsum, Zöe Borrill, Jørgen Vestbo, Dave Singh

**Affiliations:** 1 University of Manchester, North West Lung Research Centre, University Hospital of South Manchester Foundation Trust, Wythenshawe, Manchester, M33 9LT, UK

## Abstract

**Background:**

Airway inflammation in COPD can be measured using biomarkers such as induced sputum and Fe_NO_. This study set out to explore the heterogeneity of COPD using biomarkers of airway and systemic inflammation and pulmonary function by principal components analysis (PCA).

**Subjects and Methods:**

In 127 COPD patients (mean FEV_1 _61%), pulmonary function, Fe_NO_, plasma CRP and TNF-α, sputum differential cell counts and sputum IL8 (pg/ml) were measured. Principal components analysis as well as multivariate analysis was performed.

**Results:**

PCA identified four main components (% variance): (1) sputum neutrophil cell count and supernatant IL8 and plasma TNF-α (20.2%), (2) Sputum eosinophils % and Fe_NO _(18.2%), (3) Bronchodilator reversibility, FEV_1 _and IC (15.1%) and (4) CRP (11.4%). These results were confirmed by linear regression multivariate analyses which showed strong associations between the variables within components 1 and 2.

**Conclusion:**

COPD is a multi dimensional disease. Unrelated components of disease were identified, including neutrophilic airway inflammation which was associated with systemic inflammation, and sputum eosinophils which were related to increased Fe_NO_. We confirm dissociation between airway inflammation and lung function in this cohort of patients.

## Background

Chronic obstructive pulmonary disease (COPD) is an inflammatory airway disease characterised by poorly reversible airway obstruction. In fact, COPD can be viewed as an umbrella term that encompasses a range of pulmonary and systemic manifestations. COPD severity is graded by forced expiratory volume in 1 second (FEV_1_) [1], but this grading does not recognise the range of pathophysiological abnormalities that may be present in this heterogeneous condition. There is currently much interest in improving the phenotypic description of COPD by the use of biomarkers that allow distinct subgroups of patients with different prognosis or response to therapy to be identified [2].

Induced sputum is a safe and non-invasive method for studying biomarkers of airway inflammation in COPD patients, neutrophil [3] and eosinophil [4] numbers being the most valuable measures at present. An alternative biomarker is nitric oxide (NO), which is synthesized from L-arginine by nitric oxide synthase (NOS) enzymes and can be measured in exhaled breath (Fe_NO_). Fe_NO _(fractional exhaled nitric oxide) has not become widely used as a biomarker in COPD patients as it is reduced by current cigarette smoking [5] and can therefore mainly be used in ex-smokers [6,7] and subjects with unstable disease [8].

Biomarkers of airway inflammation, such as induced sputum and Fe_NO_, clearly have the potential to define subgroups of COPD patients with different characteristics. In order to use these biomarkers to enhance phenotype description, it would be important to know other clinical characteristics associated with these biomarkers. For example, patients with COPD have increased levels of systemic inflammation [9,10], with CRP levels being associated with increased mortality [11], possibly through cardiovascular disease [10]. Such associations between airway and systemic inflammation may point towards specific pathophysiological mechanisms that contribute to disease characteristics.

Multivariate modelling overcomes has been used to test pre-determined hypotheses concerning the relationships between biomarkers and other measurements in COPD [12,13]. An alternative strategy is to use methods that generate hypotheses rather than test pre-determined hypotheses. Exploratory factor analysis [14,15] is a hypothesis generating method that identifies groups of associated parameters into factors that are responsible for disease heterogeneity. This approach has previously been used in COPD to demonstrate dissociation between airway inflammation and pulmonary function [16]. Principal components analysis (PCA) is the commonest form of factor analysis and reduces a large number of variables to a much smaller number of components, explaining the variability within the data set. These components represent latent processes which cannot be directly measured. In the context of COPD, components may represent the pathophysiological processes responsible for disease heterogeneity.

We report the use of PCA to explore the heterogeneity in markers of airway and systemic inflammation and pulmonary function in a cohort of subjects with COPD. The primary aim of this study was to identify components representative of the different pathophysiological processes and hence generate hypothesis concerning COPD phenotype description. We also used traditional multivariate modelling to test the predetermined hypothesis that the non-invasive airway biomarkers studied were associated with other disease parameters.

## Methods

### Subjects

127 COPD patients (44 smokers and 83 ex-smokers) diagnosed according to current guidelines [1] with a significant smoking history (> 10 pack years), and spirometric measurements of post bronchodilator forced expiratory volume in 1 second (FEV_1_) < 80% and FEV_1_/forced vital capacity (FVC) < 0.7 were recruited. Patients were recruited from primary care by media advertising. Only subjects who had negative skin prick tests to three allergens (house dust mite, grass pollen and cat hair; ALK Abello; Denmark) were included and patients with a clinical history of asthma or atopy were excluded. Additional exclusion criteria were a respiratory tract infection or exacerbation of COPD in the preceding six weeks. The demography of all participants is shown in Table 1. Written and informed consent was obtained and the local ethics committee approved the study.

**Table 1:** Subject demography and descriptive data of variables

Variable	Mean (SD)
Age (years)	64.6 (7.6)
Gender (M/F)	80/47
ICS use (yes/no)	73/54
ICS daily dosage (microgrammes)$	990.4 (695.2)
Smoking pack years	47 (23)
IC (litres)	2.2 (0.6)
FEV_1 _(% Predicted)	61.2 (15.0)
Reversibility (%)	6.1 (5.7)
BMI	27 (0.5)
Fe_NO _(ppb)	15.9 (13.8–18.3)*
Sputum IL8 (pg/ml)	641.3 (536.5–766.6)*
CRP (mg/ml)	3.1 (2.5–3.8)*
TNFα (pg/ml)	1.6 (1.5–1.8)*
Sputum TCC (×10^6^)	4.8 (3.5–6.2)*
Sputum Neutrophil TCC (×10^6^)	4.2 (2.8–5.5)*
Sputum Eosinophil TCC (×10^6^)	0.2 (0.1–0.3)*
Sputum Neutrophil %	76.9 (73.5–80.3)*
Sputum Eosinophil %	6.2 (3.9–8.6)*

*Geometric mean (95% confidence interval presented for variables that were log transformed.

ICS; inhaled corticosteroid, IC; inspiratory capacity, FEV_1_; forced expiratory volume in 1 second, BMI; body mass index, Fe_NO_; fractional exhaled nitric oxide, CRP; C-Reactive Protein, TNFα tumour necrosis factor-alpha, TCC; total cell count.

$ Beclomethasone dipropionate equivalent ICS total daily dosage in 73 ICS users

### Study design

The following procedures were performed on a single study visit in order: measurement of Fe_NO_, spirometry, plethysmography, sputum induction and peripheral blood sampling. Inhaled corticosteroids were withheld for 12 hours prior to the study visit.

#### Fe_NO_

Subjects were asked to abstain from food and caffeine for two hours, nitrate enriched foods for 24 hours, smoking for six hours, and alcohol for twelve hours prior to the measurement of Fe_NO _using a Niox chemiluminescence on-line analyser (Aerocrine, Solna, Sweden). The smoking history was checked by questioning before Fe_NO _measurements were commenced. After inhaling NO free air to total lung capacity, subjects exhaled at a constant flow rate against a resistor to collect the plateau NO concentration at flow rate 50 ml/s (ATS guidelines). Three acceptable readings were recorded according to the American Thoracic Society guidelines [17].

#### Pulmonary function

Maximum expiratory flow volume measurements were performed in triplicate using the spirometry system on the Masterscreen; we recorded the highest FEV_1 _and FVC. Readings were repeated 15 minutes after 200 mcg Salbutamol via spacer. Inspiratory capacity (IC) was measured in a constant volume plethysmograph (Sensormedics Vmax 6200).

#### Induced sputum

Sputum was induced using 3%, 4% and 5% saline, inhaled in sequence for 5 min via an ultrasonic nebuliser (Ultraneb 2000, Medix, Harlow, UK). Sputum was selected from the saliva, and processed with DTT as previously described [18]. Cytospin preparations were air dried, fixed with methanol and stained with Rapi-diff (Triangle, Skelmersdale, UK). Four hundred leukocytes were counted and the results expressed as a percentage of the total leucocyte count, and a total cell count (TCC).

#### Sputum supernatant cytokine analysis

Interleukin 8 (IL-8) was measured by enzyme linked sandwich immunoassay (ELISA) (R&D Systems Europe, Oxon, UK) with a lower limit of detection of 15.625 pg/ml.

#### Plasma assays

Plasma was obtained from peripheral blood samples by centrifugation at 2500 rpm and 4°C for 15 minutes. Plasma was stored at -80°C until analysis. Tumour Necrosis Factor-alpha (TNF-α) was measured by high sensitivity ELISA (Quantikine, R&D Systems Europe, Oxon, UK) with a lower limit of detection of 0.5 pg/ml. C-reactive protein (CRP) was measured by high sensitivity particle enhanced immunonephelometry (Cardiophase; BN systems, Dade Behring, Newark, USA) with a lower limit of detection of 0.175 mg/L.

### Statistical analysis

All statistical analyses were performed using SPSS 13.0 (SPSS Inc, Chicago, Ill). The Kolmogorov Smirnov test determined normality of data. Non-parametric data were natural log transformed and presented as geometric means and 95% confidence intervals. Statistical significance was considered at p ≥ 0.05. PCA analysis was performed as follows:

#### 1. Variable selection

The following variables were included: FEV_1 _(% predicted), IC (L), reversibility (% predicted), Fe_NO _(ppb), CRP (mg/L), TNF-α (pg/ml), sputum TCC (×10^6^), sputum neutrophil TCC (×10^6^) and eosinophil % and sputum IL8 (pg/ml). The PCA was not run with sputum neutrophil % and eosinophil % as they are mathematically related. Instead, neutrophil TCC was included as this reflects the neutrophil load in the airways.

#### 2. Component extraction

We interpreted only the loadings with an absolute value greater than 0.4 (which explains around 16% of the variance by the variable) [15]. Missing data cases were excluded pair wise rather than list wise to maintain sufficient numbers for the analysis.

#### 3. Rotation

An oblique rotation was chosen based on the implausibility of independent components assumed by orthogonal rotations. However, both oblique promax and orthogonal varimax rotations were examined and produced extremely similar components demonstrating stability of the components.

#### 4. Component Validity

Component scores for each patient were calculated using the regression method. To validate the components, a MANOVA (multivariate analysis of variance) was run with the PCA scores as outcome variables and the demographic details (age, gender, smoking status, smoking pack years, BMI and inhaled steroid usage) as the predictors. If the predictor terms were significantly related to the PCA components according to Pillai's test then individual associations between predictors and components were examined using specific post hoc tests.

#### Multivariate analysis

Univariate analysis was initially performed between all variables. Those variables that were associated with more than one other variable (P < 0.2) were entered into multivariate regression models. This allowed variables that were independent predictors of the variables after adjusting for potential confounding variables to be determined. Measurements of airway inflammation (induced sputum measurements including cell counts and percentages and Fe_NO_) were the dependent variables. Linear regression was used for continuous variables. Where 2 or more independent predictors were found, analysis of the interaction between these predictors was performed.

## Results

**Figure 1:**
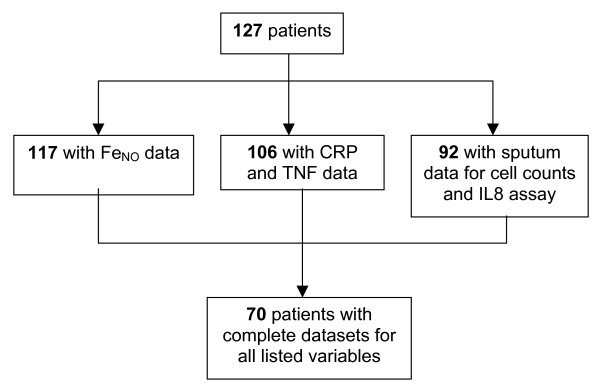
**Flow chart showing the total number of patients who were able to perform all measurements and those who were unable to complete certain measurements**.

Figure 1 shows that of the 127 patients, 10 patients could not perform Fe_NO _adequately, 21 patients did not have blood taken for analysis, and 92 produced adequate sputum for analysis. There was no difference in pulmonary function or blood biomarker measurements between the patients who could and could not perform these measurements. All patients were included in the analysis, with 70 patients having a complete dataset with all variables. The post bronchodilator FEV_1 _range for these COPD patients was 17.9 to 79.6%. 98 of the 127 patients had moderate COPD (GOLD stage II), while 22 had severe disease (GOLD stage III) and 4 very severe disease (GOLD stage IV).

### Component generation

**Figure 2:**
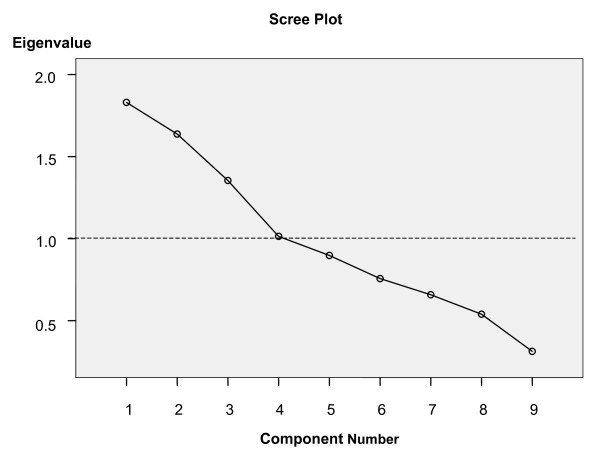
**Scree plot showing Eigenvalues for components with a reference line at Eigenvalue of 1**.

9 variables were included in the PCA (Kaiser-Meyer-Olkin measure of sampling adequacy 0.5, Bartlett's Test of sphericity < 0.0001). 4 components with eigenvalues > 1 were identified with a subsequent break in the scree plot (Figure 2). These 4 components explained 64.9 % of the variance between patients. The variables loading > 0.5 are shown in Table 2, along with the proportion of variance explained by each component.

**Table 2:** Pattern Matrix: Variable loadings for four component solution

COMPONENTS (% variance) Variables	Component Loadings			
	1	2	3	4
(1) Component 1(20.2%)				
Sputum IL8	0.79			
Sputum neutrophil TCC	0.72			
Plasma TNFα	0.76			
				
(2) Component 2 (18.2%)				
Sputum eosinophil %		0.86		
Fe_NO_		0.85		
				
(3) Component 3 (15.1%)				
FEV_1_			0.78	
Bronchodilator Reversibility			0.71	
IC			0.50	
				
(4) Component 4 (11.4%)				
CRP				0.86

All variables shown load over 0.50 onto a single PCA component.

IC; inspiratory capacity, FEV_1_; forced expiratory volume in 1 second, BMI; body mass index, Fe_NO_; fractional exhaled nitric oxide, CRP; C-Reactive Protein, TNFα tumour necrosis factor-alpha, TCC; total cell count.

*Component (1) *consisted of measurements corresponding to neutrophilic airway inflammation (sputum neutrophil cell count and sputum supernatant IL8) and systemic inflammation (plasma TNF-α) explaining the most variability in the data (20.2%). This was followed by sputum eosinophils and Fe_NO _which contributed to a similar proportion of the variance (18%), *component (2)*. A component was also formed of bronchodilator reversibility, FEV_1 _and IC measurements (15% of variance), *component (3)*. CRP levels contributed 11% of the variability, solely representing *component (4)*. The components remained unaltered when varimax rotation was applied instead of promax and even when the solution was unrotated

### Correlations between components

The correlations between the 4 components from the promax solution were weak (Table 3) showing that all the components were distinct from one another.

**Table 3:** Component Correlation Matrix

Component	1	2	3	4
1	1.000	.		
2	.001	1.000		
3	.029	.112	1.000	
4	.246	-.078	-.020	1.000

### MANOVA; Relationships between components and clinical data

Table 4 summarises the significant predictors of the components in the MANOVA. The variables in component 2 were associated with age, current smoking status and gender. Component 4 (CRP level) was associated with inhaled corticosteroid use and pack year history.

**Table 4:** Summary of MANOVA showing significant predictors of the components; predictor variables included in the analysis were age, gender, ICS use, smoking status, smoking pack years and BMI

Component	Independent Predictor	P value
1	Nil	-
2	Gender	0.04
	Smoking status	0.001
	Age	0.01
3	Nil	-
4	ICS	0.03
	Pack years	0.001

### Multivariate analysis

Different multivariate models were used to determine independent predictors of the following airway inflammation measurements; sputum total cell count, sputum neutrophil and eosinophil cell count and percentage differential and supernatant IL-8 levels (Table 5). Plasma TNF-α levels were significantly associated with sputum neutrophil cell count and supernatant IL-8 levels, and there were significant associations between sputum neutrophils and supernatant IL-8 levels. There were strong and highly significant associations (p < 0.0001) between Fe_NO _and sputum eosinophils, regardless of whether the data was expressed as percentage differential or cell count. Reversibility was associated with eosinophil percentage. Smoking and gender were independent predictors of Fe_NO _levels, with lower levels seen in COPD smokers and women. Neutrophil percentage was negatively correlated with Fe_NO _levels and reversibility.

**Table 5:** Table of results of the multivariate analyses performed to determine the independent associations between the variables

Independent Predictor	Dependent Variable						
	Fe_NO_	Sputum TCC	Sputum Neutrophil TCC	Sputum Neutrophil %	Sputum Eosinophil TCC	Sputum Eosinophil %	Sputum IL8
Fe_NO_	N/A	NS	NS	P < 0.0001 R = -0.6	P < 0.0001 R = 0.7	P < 0.0001 R = 0.62	NS
Sputum IL8	NS	NS	P = 0.002 R = 0.57	P < 0.0001 R = -0.6	NS	NS	N/A
Plasma TNFα	NS	NS	P < 0.0001 R = 0.57	NS	NS	NS	P = 0.01 R = 0.44
Smoking	P < 0.0001 R = 0.72 (lower in smokers)	NS	NS	NS	NS	NS	NS
Reversibility	NS	NS	NS	P = 0.01 R = -0.6	P = 0.02 R = 0.7	NS	NS
Gender	P = 0.04 R = 0.7 (lower in females)	NS	NS	NS	NS	NS	NS

FEV_1_, IC, BMI, Bronchodilator reversibility, plasma CRP, ICS usage and smoking pack years did not predict any of the variables marking airway inflammation.

P and R values were determined by linear regression multivariate analysis.

NS : No significant association.

TNFα; tumour necrosis factor-alpha, TCC; total cell count.

## Discussion

The primary aim of this study was to generate hypotheses about COPD phenotype description and disease mechanisms by exploring the variability in markers of inflammation and lung function using PCA. This analysis suggests that COPD is a truly multi-dimensional disease. PCA identified four main components, each explaining similar amounts of the variance (between 10 and 20%). The first two components represented neutrophilic and eosinophilic inflammation, explaining 20.2% and 18.2% of the variance respectively. Lung function parameters formed a separate component, comprising measures of airflow obstruction and reversibility. CRP also formed a separate component. Some hypotheses about disease mechanisms can be generated from this analysis; component 1 suggests that the profile of neutrophilic airway inflammation is associated with systemic inflammation, and component 2 suggests that patients with sputum eosinophilia, which is associated with increased corticosteroid responsiveness [4] also have increased Fe_NO _levels. Importantly, PCA indicates that these are distinct components of disease that could be used for patient phenotyping [19]. Correlations between the components were weak despite the use of a Promax rotation. To validate the PCA components, we performed multivariate modelling, which confirmed our PCA findings.

The main limitation of any PCA is the selection of variables included. This analysis has focused on a selection of well studied markers of airway [3,4] and systemic inflammation [13,20,21] as well as pulmonary function, by which COPD is classically defined [1]. Other important biomarkers of COPD pathophysiology, explaining further disease heterogeneity may not have been included. Nevertheless, our study shows the potential utility of PCA, and further studies using other biomarkers of inflammation or clinical measurements would be of interest.

The results of PCA are critically dependent on the selection of subjects. If particular subgroups of patients are included or excluded from the study, the sources of variation in the dataset will be affected. A common issue in studies of airway sampling in COPD patients, either by induced sputum or Fe_NO_, is that not all patients can complete each measurement [16]. We used a well validated approach to this issue, excluding cases pairwise where data was missing [14], so that all of the 92 patients with induced sputum data and 117 patients with Fe_NO _data could be analysed where possible e.g. all of these data could be analysed against pulmonary function. We did not exclude patients who could not perform certain analysis, as this may have introduced a bias into the dataset; e.g. certain patient phenotypes may produce less sputum than others, and by excluding such patients any such a phenotype would be poorly represented in the data set.

Factor analysis/PCA has been rarely used in COPD [16,22-25]. The sample for the currents study compares very favourably with these studies, which have often enrolled less than 100 subjects [22-25]. Indeed, even if we accounted for the incomplete measurements in the current study, the sample size of patients with a "complete dataset" (n = 70), is still larger than the enrolled sample size of some of these studies [16,22-25].

Recently, factor analysis has been used by Lapperre et al [16] in 114 COPD patients to generate hypothesis about disease description using many of the same parameters as the current study, but importantly systemic inflammation biomarkers were not investigated. A four factor solution was reported, with the character of the components being somewhat different to our findings. Firstly, a factor representing asthma like parameters (i.e. reversibility, bronchial hyper-reactivity and atopy) was identified. This may be due to some extent to differences in subject selection, as in our cohort subjects with asthma may have been more rigorously excluded, as atopic subjects were not recruited. Secondly, Lapperre et al reported a component including sputum percentage neutrophils and eosinophils, and that Fe_NO _was not in the same component as sputum eosinophils, which differs from our results. Important methodological issues should be considered; (a) Lappere et al used 2 different Fe_NO _analysers, but it is known that data from different analysers generates significantly different NO levels [26]. This could explain why Fe_NO _was in a distinct component, as associations with other parameters were not possible as the absolute Fe_NO _values were actually mostly dependent on the type of analyser used rather than any patient characteristics (b) Laperre et al included both sputum eosinophil and neutrophil % in the factor analysis, which together were found to form a distinct component. However, there is a mathematical relationship between these parameters (as one increases, the other decreases). The impact on PCA analysis is that the mathematical relationship between sputum percentages will cause these parameters to be associated within the same component, and may cause associations with other parameters, such as Fe_NO_, to be overlooked. We used sputum total neutrophil cell count and sputum eosinophil percentage in the PCA to avoid this issue. Reassuringly, our PCA findings concerning Fe_NO _and eosinophils were confirmed by multivariate modelling showing a significant association between these parameters.

The positive relationship between Fe_NO _and sputum eosinophils has been observed in a smaller COPD group [27] but not by Siva et al [28] in 83 COPD patients. Again, measurement methodology may give an explanation for the lack of positive findings, as Siva et al used a flow rate of 250 ml/s, which is well known to give very low Fe_NO _readings, particularly in COPD patients, and so may not be able to discriminate between patients.

Component 1 suggests that sputum neutrophils and the neutrophil chemoattractant IL-8 describe a distinct component of disease that is associated with systemic inflammation, measured by plasma TNFα levels. It is perhaps surprising that the other systemic inflammation biomarker that we measured, CRP, was not associated with airway neutrophils. CRP is a known marker of cardiovascular disease risk [29] and levels in COPD are associated with mortality [11], leading to the hypothesis that CRP levels are indicative of cardiovascular disease risk in COPD patients. A possible explanation for our findings is that neutrophilic airway inflammation is associated with some systemic inflammation pathways, such as TNFα which is known to be involved in muscle inflammation [20], but not CRP which is an indicator of cardiovascular disease.

Inhaled corticosteroid use was associated with CRP levels; this may be due to more severe patients with higher CRP levels being prescribed inhaled corticosteroids. Inhaled corticosteroid use was not associated with pulmonary function (component 3); this may be viewed as a surprising finding as corticosteroids are used for patients with lower FEV_1 _values who have exacerbations. The reason for the lack of an association in the current study was probably that the range of FEV_1 _values was relatively narrow, as most patients had moderate COPD (GOLD stage 2), and that the inclusion of greater numbers of severe/very severe patients would be needed to assess this relationship further.

It has been reported that sputum neutrophil counts [30] and IL-8 [31] levels are related to severity of airflow obstruction and subsequent decline in FEV_1 _in COPD. Airway tissue immunohistochemistry studies clearly show that mucosal inflammation is associated with lower FEV_1 _[32]. However, in agreement with the previous study by Laperre et al we found dissociation between airway inflammation and pulmonary function. This suggests that luminal inflammation, sampled by induced sputum, is not associated with FEV_1_. A strength of the current study in coming to this conclusion is the sample size used, and 2 independent statistical analysis techniques. However, it is possible that other population groups including more patients with very severe COPD may generate different results.

Our study population was composed of mainly GOLD stage 2 "moderate" COPD patients, although 26 severe/very severe patients were also recruited. This mix of patients reflects our strategy of recruiting from primary care. It would be of interest to repeat the current study using more severe patients, perhaps recruited from hospital clinics, to observe whether the same or different results are obtained.

We found that females with COPD had lower levels of Fe_NO _than males, which has been shown to be true in healthy controls [33]. Similarly, the associations of Fe_NO _with age and with smoking that we observed have also previously been reported [34].

In summary, this study provides insights into the dimensions of COPD that can be described using non-invasive biomarkers of airway inflammation and pulmonary function. Independent components representing different types of airway inflammation, lung function and systemic inflammation have been identified providing novel concepts with regards to COPD pathophysiology. We hope that our results will provide an impetus to further explore the usefulness of these components in guiding clinical practice by assessing their ability to predict important features of COPD such as prognosis, systemic manifestations and treatment responses.

## Competing interests

The authors declare that they have no competing interests.

## Authors' contributions

KR conceived, designed and coordinated the study as well as performing a lot of the measurements in exhaled NO, spirometry, plethysmography, sputum induction and processing, peripheral blood sampling and statistical analysis. DS and JV helped to conceive the study and participated in its design. JS participated in the statistical analysis. UK performed sputum induction, sputum supernatant cytokine analysis and assays for CRP and TNF-alpha. ZB performed measurements in spirometry, plethysmography and peripheral blood sampling.

All authors read and approved the final manuscript.
